# Supplementary figures and images for: Dugong dugon feeding in tropical Australian seagrass meadows: implications for conservation planning
Source: PeerJ. 2016 Jul 7;4:e2194. doi: 10.7717/peerj.2194 (PMC4941767; doi:10.7717/peerj.2194)

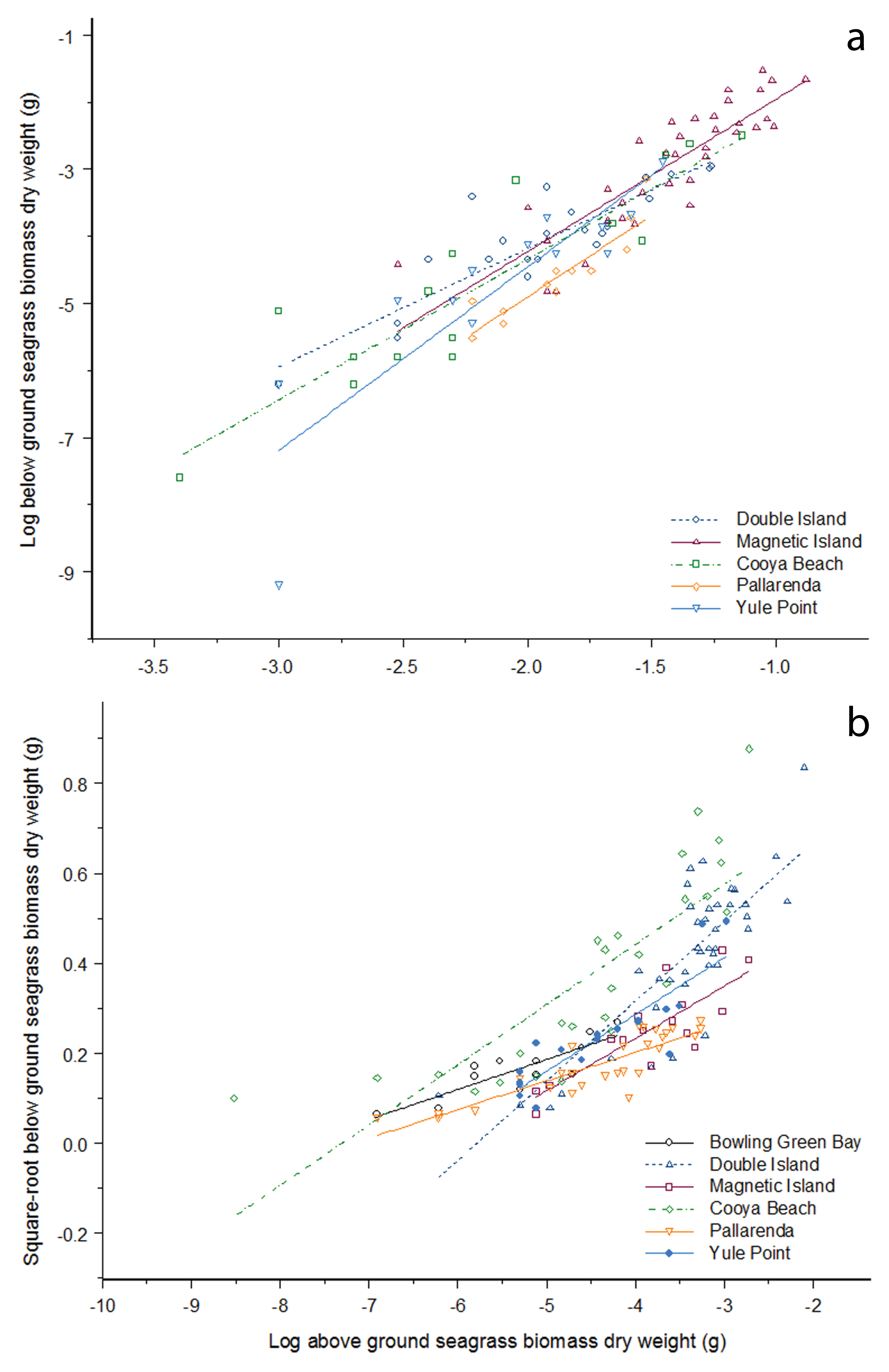

Supplement: Appendix S1 [file peerj-04-2194-s001.png]
