# Supplementary material for: Dugong dugon feeding in tropical Australian seagrass meadows: implications for conservation planning
Source: PeerJ. 2016 Jul 7;4:e2194. doi: 10.7717/peerj.2194 (PMC4941767; doi:10.7717/peerj.2194)
Supplement: Appendix S2 [file peerj-04-2194-s002.docx]

**Appendix 2:** Linear (LM) and Generalized Least-Squares (GLS) regression results for predicting below ground biomass from above ground biomass of seagrass species taken from six sites within the Great Barrier Reef, north-east of Australia.

| Site | Model | Intercept (a) | Slope (b_1_) | Slope (b_2_) | R^2^ | df | F/t statistic | p value |
| --- | --- | --- | --- | --- | --- | --- | --- | --- |
| Double Is. |  |  |  |  |  |  |  |  |
| *Cymodocea serrulata* | LM | 0.0778 | 0.6127 | NA | 0.37 | 1, 12 | 7.14 | 0.0204 |
| *Halophila ovalis* | LM | 0.0075 | 0.8552 | NA | 0.74 | 1, 22 | 60.97 | > 0.001 |
| *Halodule uninervis* | GLS | - 0.0330 | 2.3336 | NA | 0.95 | 39, 37 | 9.36 | > 0.001 |
| *Syringodium isoetifolium* | GLS | 0.0076 | 3.1578 | NA | 0.81 | 12, 10 | 5.99 | 0.0005 |
| *Thalassia hemprichii* | GLS | 0.2173 | - 3.7722 | NA | 0.89 | 8, 6 | - 2.00 | 0.0936 |
| Yule Point beach |  |  |  |  |  |  |  |  |
| *Halophila ovalis* | LM | 0.0054 | 1.0626 | NA | 0.84 | 1, 11 | 56.23 | > 0.001 |
| *Halodule uninervis* | LM | 1.5129 | 1.0518 | NA | 0.73 | 1, 15 | 39.99 | > 0.001 |
| Cooya beach |  |  |  |  |  |  |  |  |
| *Enhalus acoroides* | GLS | - 0.5791 | - 0.9570 | NA | 0.93 | 12, 10 | - 1.25 | 0.2390 |
| *Halophila ovalis* | LM | - 0.1519 | 0.0911 | NA | 0.80 | 1, 15 | 58.00 | > 0.001 |
| *Halodule uninervis* | LS | - 0.0061 | 9.6187 | NA | 0.75 | 26, 24 | - 0.24 | > 0.001 |
| *Zostera muelleri* | LM | 0.5319 | 0.5633 | NA | 0.80 | 1, 14 | 15.53 | > 0.001 |
| Cockle Bay, Magnetic Is**.** |  |  |  |  |  |  |  |  |
| *Cymodocea serrulata* | GLS | - 0.0074 | 1.5376 | NA | 0.91 | 13, 11 | 5.25 | 0.0003 |
| *Halophila ovalis* | GLS | - 0.0005 | 1.5800 | NA | 0.96 | 36, 33 | 6.82 | > 0.001 |
| *Halodule uninervis* | GLS | 0.0077 | 2.4301 | NA | 0.85 | 16, 14 | 4.57 | 0.0004 |
| Cape Pallarenda |  |  |  |  |  |  |  |  |
| *Halophila ovalis* | LM | - 0.0179 | 1.0613 | NA | 0.83 | 1, 12 | 59.86 | > 0.001 |
| *Halodule uninervis* | GLS | 0.0151 | 1.3300 | NA | 0.94 | 28, 26 | 9.01 | > 0.001 |
| Bowling Green Bay |  |  |  |  |  |  |  |  |
| *Halodule uninervis* | GLS | 0.0036 | 4.2921 | NA | 0.84 | 12, 10 | 5.82 | 0.0002 |
| *Zostera muelleri* | LM | - 6.2533 | - 2.5475 | - 0.4098 | 0.59 | 2, 23 | 16.41 | > 0.001 |
